# The quality of life and body image disturbances of Turner syndrome patients in Malaysia: a cross-sectional study

**DOI:** 10.1186/s12905-023-02745-x

**Published:** 2023-11-17

**Authors:** Amirul Ashraf Bin Yusof, Manuela Lee Sze Chii, Nur Izzarizlyn Mohammad Yusoff, Raja Nur Iman Farhani Raja Mazrul Kama, Jairus Reuben Raj, Nur Azurah Abdul Ghani, Anizah Ali, Joyce Hong Soo Syn, Shamsul Azhar Shah, Nurkhairulnisa Abu Ishak, Adongo Susan Akinyi, Ani Amelia Zainuddin

**Affiliations:** 1 Department of Obstetrics and Gynaecology, Faculty of Medicine, The National University of Malaysia (UKM), Jalan Yaacob Latif, Bandar Tun Razak, Cheras, 56000 Kuala Lumpur, Malaysia; 2 Department of Paediatrics, Faculty of Medicine, The National University of Malaysia (UKM), Jalan Yaacob Latif, Bandar Tun Razak, Cheras, 56000 Kuala Lumpur, Malaysia; 3 Department of Community Health, Faculty of Medicine, The National University of Malaysia (UKM), Jalan Yaacob Latif, Bandar Tun Razak, Cheras, 56000 Kuala Lumpur, Malaysia; 4 Department of Obstetrics and Gynaecology, Kulliyyah of Medicine, Indera Mahkota Campus, International Islamic University Malaysia, 25200 Kuantan, Pahang Malaysia; 5 Department of Obstetrics and Gynecology, Division of Surgery, Kenyatta National Hospital, University of Nairobi, Nairobi, Kenya

**Keywords:** Turner syndrome, Quality of life, Body image disturbance, Social relationship

## Abstract

**Background:**

Turner Syndrome (TS) is a rare sex chromosome abnormality occurring in 1 in 2500 female live births. To date, there is limited data on TS patients in Malaysia. This study aimed to investigate the quality of life (QoL) and body image disturbances among adult population with TS in comparison to age-matched controls in a tertiary hospital in Kuala Lumpur: Hospital Chancellor Tuanku Mukhriz, Universiti Kebangsaan Malaysia (HCTM, UKM).

**Methods:**

This was a cross-sectional study carried out in HCTM, UKM, Kuala Lumpur. TS participants who attended clinic in HCTM, UKM and controls who were hospital staff members were recruited via purposive sampling. TS participants’ sociodemographic and clinical profiles were retrieved from medical records. Two validated, translated questionnaires; World Health Organization Quality of Life (WHOQOL-BREF) questionnaire and Body Image Disturbances Questionnaires (BIDQ) were completed by participants.

**Results:**

A total of 34 TS patients were approached and 24 (70.5%) of them participated in this study. Their median (IQR) age was 24.0 (7.0) years and their responses were compared to 60 age-matched healthy females as controls [median age (IQR) = 24.0 (8.0) years]. The most common medical problem in TS participants was premature ovarian insufficiency (*n* = 23; 95.8%). There were no significant differences between TS and control groups’ median scores (overall QOL; 4.00 vs. 4.00, general health; 3.50 vs. 4.00, physical health; 14.86 vs. 15.43, psychological health; 14.67 vs. 14.00 and environment; 15.00 vs. 15.50) of the different WHOQOL-BREF domains. However, TS participants were found to score 13.33 against 16.00, lower than the control group (*p* < 0.05) in the social relationship domain. Comparatively, body image concerns among TS respondents were significantly higher in impairment in the mainly social areas of functioning (*p* < 0.05).

**Conclusion:**

The study demonstrated that the overall QoL of TS participants was good and almost similar to that of the controls. However, TS group had significantly lower scores for social domain and had greater concerns in social interactions, thus affecting their social life.

## Background

Turner syndrome (TS) is a sex chromosome abnormality, which results from partial or complete loss of one of two X chromosomes affecting approximately one out of every 2500 female live births [1]. The affected females have a genetic profile of either 45 XO karyotype, known as the classical Turner karyotype which account for 50% of cases or the mosaic karyotype; 45 XO/46 XX, 45 XO / 46 XY or even 45 XO / 47 XXX or 45 XO/ 46 XX with an abnormality in the X chromosome. The usual triad of clinical features that are often seen in patients with this syndrome are short stature, webbed neck due to the distended lymphatic channels and typical facies including micrognathia and low posterior hairline [2]. Cardiovascular abnormalities such as coarctation of aorta is the most common cause of mortality in childhood whilst in adolescence, affected girls fail to develop normal secondary sex characteristics whereby they have no breast development, infantile genitalia and absence or reduced pubic hair. The cognitive function of these patients is usually normal but they may have subtle defects in non-verbal, visual-spatial information processing [3]. According to Morris et al. [4], the findings of the study indicate that individuals with TS may have an equal or greater risk for depression or depressive symptoms than those unaffected by TS. The risk may be more prominent as an individual reaches the age of adulthood.

Research regarding TS patients’ life quality especially in the South East Asian region are scarce, with no known study yet to be conducted in Malaysia. This research is thus an initiative to understand the impact of the syndrome on the patients living with this lifelong and often complicated conditions. Expanding established research in this elusive study topic could help the healthcare providers to improve on various aspects of the service that are deficient. It is worrying that parents of TS patients have reported that their children are socially inept with having few friends when compared to their peers and spend significantly less time with their friends [5]. In addition, Morris et al. [4] study findings strongly suggest that clinical research on depression risk in girls and women with TS is lacking, which could relate to the inadequate mental health care for this community. Therefore, we aim to fill in these gaps with this study.

A subjective, multifaceted notion, quality of life (QoL) stresses a person's perception of their own condition of affairs at any given time which includes social and psychological well-being as well as health status (such as morbidity). When referring to QoL as it relates to illnesses or treatments that people go through, the term "health-related quality of life" is frequently used. In contrast, a person's assessment of their overall quality of life includes how safe their environment is, how easily they can obtain medical care and social services, and how they feel about their current spiritual situation [6]. The World Health Organization Quality of Life (WHOQOL) group, a global research organization, defines quality of life as an individual's perception of their place in life in relation to their goals, expectations, standards, and concerns, as well as the culture and value systems in which they live [6].

As such, an instrument was developed by World Health Organisation in order to have better assessment that encompasses an individual life quality holistically. The domains identified include six broad domains which describe core aspects of quality of life cross-culturally: a Physical domain (e.g., energy and fatigue), a Psychological domain (e.g., positive feelings), Level of independence, (e.g. mobility), Social relationships (e.g., practical social support), Environment (e.g., the accessibility of health care), and Personal beliefs/Spirituality (e.g., meaning in life), henceforth giving rise to the WHOQOL-100 with 100 items were selected for inclusion in the questionnaire. These included four items for each of 24 facets of quality of life, and four items relating to the Overall Quality of Life and General Health facet [7].

The WHOQOL-100 enables a thorough evaluation of every aspect of quality of life. But occasionally, the WHOQOL-100 might be too long for actual use. One item from each of the 24 facets found in the WHOQOL-100 has been included to provide a broad and thorough assessment. In addition, two items from the facets of general health and overall quality of life have been added leading to the more comprehensive tool, WHOQOL-BREF with 26 items instead of 100 [8].

In Malaysia, due to the lack of public awareness of this condition, poor sex education in schools that educate on puberty changes and what is normal / abnormal as well as the cultural influences, these patients fail to get early medical attention. Not all healthcare providers are well versed on this condition. Therefore, when patients do seek help, they may not get adequate or appropriate care. The possibilities of the cultural influences, public acceptance, knowledge and healthcare services provided may also be factors which affect these patients’ quality of life. The study aims to investigate the quality of life and body image disturbances of adult patients with TS attending a tertiary centre in Malaysia.

## Methods

Participants in this cross-sectional study were genetically female patients with TS, aged > 18 years and raised female. Databases from a tertiary referral centre in Malaysia namely the Paediatric Adolescent Gynaecology (PAG) Unit and Paediatric Endocrine Clinic in Hospital Chancellor Tuanku Mukhriz, Universiti Kebangsaan Malaysia (HCTM, UKM) were searched to identify potential participants. Those who attended the PAG or Paediatrics Endocrine clinic and fulfilled the criteria were invited to participate. There were 34 TS participants who were approached and 24 of them participated. Sixty controls were age-matched patients who were hospital staff members and students from the Faculty of Medicine of UKM.

Potential participants were informed about the study either by the researchers or their usual clinicians when they attended their clinic appointments. The researchers explained the study and obtained informed consent from those who agreed to participate. Recruitment and completion of questionnaires were carried out via Google Form or undertaken at the time of clinic appointment. Two validated, translated questionnaires; World Health Organization Quality of Life (WHOQOL-BREF) questionnaire (with 26 questions within the domains of Overall QoL, General Health, Physical Health, Psychological Health, Social Relationship and Environment) and Body Image Disturbances Questionnaires (BIDQ) which consists of 7 questions, were completed by participants.

With regards to the WHOQOL-BREF, this is an established QoL questionnaire with good to excellent psychometric properties [9]. It has low respondent burden, high acceptability and feasibility, excellent internal consistency (0.92) and good test–retest reliability. Distinctive QoL profiles were found for diverse conditions thus making it a good research tool to use for Turner syndrome patients. The Malay version of the WHOQOL BREF has also been shown to have excellent psychometric properties [10]. The scores are transformed into a linear scale between 0 and 100, where 0 is least favourable and 100 being the most favourable [11].

The BIDQ also has been shown to have good psychometric properties where it has been demonstrated to have good internal consistency, good test–retest reliability and high concurrent validity [12]. As the scoring is the mean of the responses to the items, the higher the score means the higher the concern [13].

The questionnaires, with two language choices made available in either English or Bahasa Melayu were administered via a softcopy version made on Google Forms and also using physical copies that were given to participants. This study was conducted from July 2021 till September 2021 during the Coronavirus disease 2019 (COVID-19) pandemic thus most of the questionnaires were given to participants via Google Forms as many could not attend the clinics physically due to the Movement Control Order (lockdown) implemented by the Malaysian government. The participants were given researchers’ contact information should they need assistance or faced any difficulties whilst answering the questionnaires. However, all of the participants were able to answer both sets of questionnaires without any additional help from researchers. The first part of the study describes the sociodemographic and clinical profiles of the study cohort, while the second part compares the quality of life [6, 8] and body image disturbances (BID) [14] of the TS patients with the age-matched healthy control group. Medical records were reviewed. Approval for the use of these questionnaires were obtained from the respective authors.

### Sample size

The rarity of the conditions as well as the relatively low number of diagnosed and scarcity in the local reported cases have in turns made finding large and great number of TS patients difficult. However, HCTM UKM is one of the largest tertiary centres with TS patients in the databases, thus a purposive sampling was used in acquiring the required number of TS patients as needed.

The sample size is calculated using PS Power and Sample Size Calculations Version 3.1.2 software. The values input include α; the type I error probability for a two sided test which is the probability that will falsely reject the null hypothesis, power; the probability of correctly rejecting the null hypothesis of equal population means, δ; the difference in population, σ; the group standard deviation and m; the ratio of control to experimental patients. These are the parameters that are input into the software to calculate the appropriate sample size for this study.

This study is planned to be of a continuous response variable from independent control and TS subjects with the value of m is 2.5:1 meaning 2.5 controls per TS subject. The ratio is chosen to have higher number of control study against TS patients in order to obtain a sample size that is suitable enough for the study especially since our database showed that we have 34 TS patients and accounting for those who may refuse to participate, therefore more control subjects among the healthy populations is chosen. In the study by Liedmeier A. et al. [15], the response within each TS patient was normally distributed with standard deviation, σ of 16.81. We have taken this study result as their study shows the latest result for the QOL in TS patients using WHOQOL-BREF instrument which is similar to this study.

Next, the δ value calculated which is the true difference in the TS patients in Liedmeier A. et al. [15] in comparison to the healthy population means score in Hawthorne G. et. al [16]. for WHOQOL-BREF is 10, thus we will need to study 24 TS subjects and 60 control subjects to be able to reject the null hypothesis that the population means of the experimental and control groups are equal with probability, power of 0.7. The value of α which is type I error probability associated with this test of this null hypothesis is 0.05. The total number of participants is therefore 84 people.

### Statistical analyses

All statistical analyses were performed using IBM Statistical Package for Social Sciences version 26.0. Descriptive analyses were carried out for the TS respondents which included mean ± standard deviation (SD), median, interquartile range (IQR), ranges for the continuous variables. Frequencies and percentages were used for categorical variables. Normality of the distribution of data was determined by the Shapiro–Wilk tests. Comparison of specific outcomes of the TS group with age-matched controls, for continuous variables, utilised the Mann–Whitney U tests for abnormally distributed data. For categorical variables, Pearson’s chi-square tests and Fisher’s Exact tests for association were used and a *p*-value of less than 0.05 taken to indicate statistical significance.

## Results

### Sociodemographic and clinical profiles

Thirty-four potential participants were identified and out of these, 24 consented to participate. The sociodemographic details of the TS participants are tabulated in Table 1. The median age (IQR) among TS participants was 24(7) years (range 19–49 years). There was a significant difference in ethnicity, living with, employment status and monthly income group. The controls had a higher percentage in marital status (married) and family income. However, they had a lower percentage in the aspect of living with parents. Among women with TS, 20 (83.3%) of them lived with their parents. Only one TS participant was married. Six (25%) of women with TS in our study were neither employed nor a student. Most TS participants came from families with average monthly incomes that were from the lowest income bracket for Malaysia, the B40 group.

**Table 1:** Sociodemographic background of TS patients and control group

Variables	TS Patients (*n* = 24)		Control Group (*n* = 60)		*p*-value
**Age (Years)**					0.866^#^
Median (IQR)	24 (7)		24 (8)		
Range	19—49		20—50		
	n	%	n	%	0.045^*^
**Ethnicity**					
Malay	16	66.7	30	50.0	
Chinese	8	33.3	17	28.3	
Others	0	0	13	21.7	
**Religion**					0.587^*^
Islam	16	66.7	33	55.0	
Buddhist	5	20.8	15	25.0	
Others	3	12.5	12	20.0	
**Living With**					0.029^*^
Parents	20	83.3	35	58.3	
Others	4	16.7	25	41.7	
**Marital Status**					0.097^$^
Single	23	95.8	48	80.0	
Married	1	4.2	12	20.0	
Divorced/Separated	0	0	0	0	
Widowed	0	0	0	0	
**Highest Level of Education**					1.000^$^
No education	0	0	0	0	
Primary education	0	0	0	0	
Secondary education	2	8.3	5	8.3	
Tertiary education	22	91.7	55	91.7	
**Employment**					0.002^*^
Unemployed and not a student	6	25	1	2.1	
Student	10	41.7	39	64.6	
Employed	8	33.3	20	33.3	
**Monthly Income Group**					0.043^*^
B40	16	66.7	22	36.7	
M40	6	25	30	50.0	
T20	2	8.3	8	13.3	

Significant at the level of 0.05 level

*TS* Turner syndrome, *IQR* interquartile range involving patients, *B40* below 40%, *M40* middle 40%, *T20* top 20%

^#^ Mann–Whitney U Test; ^*^ Chi-Square Test; ^$^ Fisher’s Exact Test

Table 2 shows the clinical profiles of the TS participants. The mean age at diagnosis (± SD) was 16.75 ± 5.22 years (range:6–27). The majority of TS participants were classical TS (58.3%). The mean height of our TS participants (± SD) was 148 ± 9.21 cm. The most common medical problem among TS participants in our study was premature ovarian insufficiency (95.8%), followed by osteopenia or osteoporosis (62.5%).

**Table 2:** TS patients’ clinical profile

Variables		
**Age at Diagnosis (Years)**		
Mean ± SD	16.75 ± 5.219	
Range	6–27	
	n	%
**Type of TS**		
Classical	14	58.3
Mosaic	10	41.7
**Medical Problems**		
Premature ovarian insufficiency	23	95.83
Osteopenia/Osteoporosis	15	62.5
Hearing	9	37.5
Metabolic dysfunction	6	25.0
Scoliosis	3	12.5
Visual	5	20.8
Cardiac	2	8.33
Hypertension	1	4.16
Renal	0	0
**Number of Co-Morbidities**		
0	1	4.2
1–2	12	50.0
> 2	11	45.8

*TS* Turner Syndrome

### Comparing the overall QoL and general health between adult TS participants and controls

Fisher’s exact tests were used to compare the overall QoL and general health by rating scale of TS patients and age-matched control group. Of the 84 participants (controls and TS patients), none scored their overall QoL as very poor or poor. Also, none of the participants were very dissatisfied with their general health. There was no significant difference between the overall QoL of TS participants and non-TS participants. There was no significant difference between the general health of TS participants and the control group participants (Table 3).

**Table 3:** Comparing overall QoL and general health by rating scale of TS patients with control group

Variables	TS Patients (*n* = 24)		Control Group (*n* = 60)		*p*-value
	n	%	n	%	
**Overall QoL**					0.587
Neutral	7	29.2	14	23.3	
Good and very good	17	70.8	46	76.7	
**General Health**					0.111
Very dissatisfied	0	0	0	0	
Dissatisfied	1	4.2	7	9.5	
Neither satisfied nor dissatisfied	11	45.8	12	27.4	
Satisfied	10	41.7	35	53.6	
Very satisfied	2	8.3	6	9.5	

*QoL* Quality of Life, *TS* Turner Syndrome

### Comparing QoL domains between adult TS participants and controls

Mann–Whitney U test was used to compare the results as the scores were not normally distributed (Table 4). There was no significant difference between the TS and control groups in all domains of QoL except for the social relationship domain. The age-matched control group participants reported a significantly higher median score for social relationship [median (IQR) = 16.00(2.67)] compared to that of the TS respondents [median (IQR) = 13.33(4), *p* = 0.04]. Social relationship in control group was significantly better than those in TS patients.

**Table 4:** Comparing median scores of WHOQOL-BREF domains between TS patients and control group

Domains	TS Patients (*n* = 24)		Control Group (*n* = 60)		*p*-value
	Median	IQR	Median	IQR	
**WHOQOL-BREF**					
Overall QoL	4.00	1	4.00	0	0.638
General Health	3.50	1	4.00	1	0.303
Physical Health	14.86	2.71	15.43	3.43	0.158
Psychological Health	14.67	2.67	14.00	2.67	0.800
Social Relationship	13.33	4	16.00	2.67	0.040*
Environment	15.00	2.75	15.50	2.50	0.128

*QoL* Quality of Life, *TS* Turner Syndrome

^*^Significant at the level of 0.05 level

### Correlations between the domains and ratings for overall QoL and general health for the TS group

In Table 5, the Spearman correlation that depicts correlations between the domains and ratings for overall QoL and general health for participants who were TS patient is shown in Table 5. All domains, physical health (*r* = 0.523) and psychological health (*r* = 0.540), social health (*r* = 0.405) and environment (*r* = 0.495) had significant positive correlation with overall QoL (*p* < 0.05). On the other hand, psychological health (*r* = 0.694) and social relationship (*r* = 0.421) were the only domain that significantly correlated with general health (*p* < 0.05) while other domains were not significant. Between the domains, all domains were significant with positive correlations, *r* = 0.528–0.738 (*p* < 0.05).

**Table 5:** Correlations between the QoL domains and rated overall QoL and general health among TS patients (*N* = 24)

	**OvQoL**	**GenH**	**PhyH**	**PsyH**	**SocR**	**Env**
OvQoL	1					
GenH	0.415^b^	1				
PhyH	0.523^a^	0.333	1			
PsyH	0.540^a^	0.694^a^	0.629^a^	1		
SocR	0.405^b^	0.421^b^	0.528^a^	0.692^a^	1	
Env	0.495^b^	0.399	0.738^a^	0.669^a^	0.641^a^	1

*OvQoL* Overall quality of life, *GenH* General health, *PhyH* Physical health, *PsyH* Psychological health, *SocR* Social relationship, *Env* Environment

^a^Correlation is significant at the level 0.01 level (2-tailed)

^b^Correlation is significant at the level 0.05 level (2-tailed)

### Correlations between the domains and ratings for overall QoL and general health for the controls

The correlations between the domains and ratings for general health and overall QoL for the control group is presented in Table 6. Physical health (*r* = 0.323), psychological health (*r* = 0.390) and environment (*r* = 0.286) were significantly correlated, with overall QoL (*p* < 0.05). There was no significant relationship between overall QoL and social relationship (*p* > 0.05). On the other hand, physical health (*r* = 0.350), psychological health (*r* = 0.496) and environment (*r* = 0.265) were the domains which significantly correlated with general health (*p* < 0.05) while social relationship was not significant. Between the domains, only environment and psychological health (*r* = 0.241) did not significantly correlate (*p* > 0.05). Others were significant with positive correlations, *r* = 0.402–0.653 (*p* < 0 0.05).

**Table 6:** Correlations between the QoL domains and overall QoL and general health among control group (*N* = 60)

	**OvQoL**	**GenH**	**PhyH**	**PsyH**	**SocR**	**Env**
OvQoL	1					
GenH	0.362^a^	1				
PhyH	0.331^a^	0.334^a^	1			
PsyH	0.410^a^	0.486^a^	0.590^a^	1		
SocR	0.221	0.241	0.402^a^	0.536^a^	1	
Env	0.266^b^	0.244	0.653^a^	0.241	0.437^a^	1

OvQoL: Overall quality of life; GenH: General health; PhyH: Physical health; PsyH: Psychological health; SocR: Social relationship; Env: Environment

^a^Correlation is significant at the level 0.01 level (2-tailed)

^b^Correlation is significant at the level 0.05 level (2-tailed)

### Comparing the BID between adult TS participants and controls

In Table 7, a total of 24 TS and 60 control participants completed the BIDQ. Pearson Chi-square was used to compare the BID between TS patients and age-matched controls. Concerns causing impairment in social, occupational, or other areas of functioning and concerns interfering with studies, job, or ability to function in role between TS patients and control group were statistically described, *p*-value < 0.05. Body image concerns among TS respondents are significantly associated with impairment in social, occupational, or other areas of functioning and interferes with studies, job, or ability to function in role compared to that of control group respondents. Mann–Whitney U test was used to compare the total BIDQ score as the scores were not normally distributed. There was no significant difference between the median scores.

**Table 7:** Comparing BID between TS patients and control group

Variables	TS Patients (*n* = 24)		Control Group (*n* = 60)		*p*-value
	n	%	n	%	
**BIDQ**					
**Concerns with unattractive body parts**					0.871
Yes	18	75.0	46	76.7	
No	6	25.0	14	23.3	
**Preoccupied with concerns of unattractive body parts**					0.725
Yes	15	62.5	35	58.3	
No	9	37.5	25	41.7	
					0.068
**Concerns causing distress, torment, or pain**					
Yes	18	75.0	32	53.3	
No	6	25.0	28	46.7	
**Concerns causing impairment in social, occupational, or other areas of functioning**					0.019*
Yes	16	66.7	23	38.3	
No	8	33.3	37	61.7	
**Concerns interfering with social life**					0.124
Yes	11	45.8	17	28.3	
No	13	54.2	43	71.7	
**Concerns interfering with studies, job, or ability to function in role**					0.041*
Yes	10	41.7	12	20.0	
No	14	58.3	48	80.0	
**Avoidance of things because of concerns**					0.727
Yes	11	45.8	25	41.7	
No	13	54.2	35	58.3	
**Median score (IQR)**	9.00 (4.00)		11.00 (4.00)		0.103^#^
**Range**	7.00–14.00		7.00–14.00		

^#^Mann Whitney U test, *Significant at the level of .05 level

## Discussion

Health-related QoL (HRQOL) is the functional effect of an illness or disorder and its consequent therapy upon a patient, as perceived by the patient [17]. Though subjective, it is a crucial aspect in patient care and provides valuable insight into holistic management of health conditions.

TS affects growth and development and its comorbidities have an impact on several facets of a patient’s life. Deeper scrutiny into the HRQOL of these patients could increase awareness regarding their needs leading to finer tailoring of their treatment process.

### Social and demographic characteristics

In our study, although not statistically significant, majority of our TS participants were single (95.8%) compared to the control group (80%). Social isolation has been more commonly reported in patients with TS than in the general population and loneliness contributes to lower QoL [18].

A higher percentage of the TS participants were neither employed nor a student which could be either due to health-related problems like hypertension as reported by Verlinde et al. [19] or body image issues [20]. Indeed, patients with TS reported the least positive overall body image compared with the other DSD groups [20] and low energy levels and fatigue [21] which might affect their ability to gain and maintain employment. There was also a feeling of being overburdened with work and concerns about working future [21]. 

### Age at diagnosis

The mean age at diagnosis (± SD) among women with TS in our study was 16.75 ± 5.22 years as compared to the study by Reimann et al. [22], where the median age at diagnosis was 12 years (0–43). In another study by Liedmeier et al. [15], the median age at diagnosis was 10.49 years (0–61). According to Bannink et al. [23] growth hormone therapy and estrogen therapy had beneficial effects and greater psychological impact on the patient’s quality of life.

### WHOQOL-BREF domains

In our study, the social dimension of QoL appear to be decreased in individuals with TS, compared to the control samples. Previous studies which used WHOQOL-BREF, to examine the QoL have found females with TS to have impairment in not only the social but also the physical and the psychological dimensions of QoL [8]. This may be because height plays an important role in the Caucasian population. Short stature is a detrimental factor which may have a tremendous effect on them psychologically as seen by the outcome of the research by Jeż et al. [18] indicating that life satisfaction can be low among TS patients concerned by short statures, in addition to also the feeling of being handicapped, their loneliness and the attitude of negative perception by those around them. On the other hand, in the Asian society, short stature is fairly common, which may also explain the delayed age at diagnosis.

There was no statistically significant difference in the general, physical, psychological health or environment aspects in both groups. These findings might be in line with the well-being paradox, which challenges the presumptions among clinicians and society that TS patients generally have poorer QoL. The correlation matrix shows interrelations within the QoL domains; however, it does not give any information on which variable can explain the global QoL best. The influence of the COVID-19 pandemic on every individual from different dimensions may have had an effect on overall QoL, general health, psychological and environmental dimensions.

### Body image disturbances

The main concerns among the TS women in our study regarding their appearance was short stature and low self-esteem – which may be a factor in the impairment of body image and social interaction. These two factors were gained from their answers to the open-ended questions in the BIDQ. Women with TS in our study have body image concerns associated with the impairment in social areas of functioning. These concerns interfered with their social life, work, job and ability to function. The interference with social functioning requiring interaction with others was suggested by Hoven et al. [21] whose TS patients in their study preferred to be involved in individual sports activities such as running or fitness presumably due to the lower satisfaction with their body image and hence, felt more comfortable in the situation of being alone. This is similar to a study by Zainuddin et al. [13] that showed women with congenital adrenal hyperplasia felt that they were not beautiful, felt different from others and embarrassed to the point of avoiding attendance to social events where social interaction with others was required.

On the other hand, previous studies by Gould et al. [24] and Liedmeier et al. [15] showed that there was no subjective impairment in the social life of women with TS.

Challenges in social interaction included connection with peers or engaging in a romantic relationship [21].

Unfortunately, there were no other studies which utilised the same instrument, BIDQ. However, another study by Cragg and Lafreniere [25], which used three instruments including the Rosenberg Self-Esteem Scale by Morris Rosenberg, the State Self-Esteem Scale by Todd F. Heatherton & Janet Polivy and the Body-Esteem Scale by Beverley K. Mendelson & Donna R. White, demonstrated that women with TS had significant lower scores on body esteem (indicating poorer body image), overall self-esteem, social and appearance-related self-esteem which parallels with our study’s findings. Our findings are also in line with the study by Wolstencroft and Skuse [5], that found that women with TS are likely to encounter social interaction challenges.

### Strengths and limitations

There is a scarcity of published research in South East Asia with regards to the QoL and BID of TS patients, hence our study findings contributed valuable additional literature to this part of the world. Our findings of the TS participants were compared with age-matched healthy control groups. Two validated, translated questionnaires were utilised in our study. Our tertiary centre, HCTM, UKM has the most established PAG unit in Malaysia, is the main referral centre, where we receive and manage the largest number of TS patients in the country.

The study however faced some limitations: It was done during the COVID-19 pandemic situation, which was far from ideal. Due to the low clinic turnout rate, it was difficult to engage with the patients face-to-face. This may have contributed to the lower participation rate. A larger study with more participants would give the research greater power with reduced bias.

We also postulate that COVID-19 situation may have contributed to the low employment rate. However, this should be investigated further.

### Recommendations

We would like to recommend a future study to compare the HRQOL in TS patients who have undergone treatment early against those who have received treatment late due to a delayed diagnosis instead of against a healthy population.

In addition, a study regarding the relationship of the TS patients with their caregivers and family members, in regards to three main stems which are depression, stress and anxiety would be useful. Findings could help in strategizing interventions to help reduce stress, anxiety and depression among caregivers of TS patients.

There is need to further screen patients with TS on varying aspects of psychosocial functioning and its impact on HRQOL since this was one of the positive findings in our study.

## Conclusion

Quality healthcare aims at improving outcomes that matter most to patients. BID with concerns causing impairment in social, occupational, or other areas of functioning which resulted in concerns with work and social environment were seen to reduce the QoL in TS patients. This may in turn result in social isolation and high unemployment rates. Improving the QoL of TS patients should therefore be one of the goals of any health care intervention such as earlier age of identification of individuals with TS via genetic testing, increasing public awareness of the conditions through mass media campaigns and reducing stigmatisation towards TS patients by formal introduction and education of the existence of the conditions among society.

## Data Availability

The datasets used and/or analysed during the current study are available from the corresponding author on reasonable request.

## Abbreviations

BID: Body image disturbances
BIDQ: Body Image Disturbances Questionnaires
COVID-19: Coronavirus disease 2019
DSD: Disorders of sex development
HCTM, UKM: Hospital Chancellor Tuanku Mukhriz, Universiti Kebangsaan Malaysia
HRQOL: Health-related QoL
IQR: Interquartile range
PAG: Paediatric Adolescent Gynaecology
QoL: Quality of Life
SD: Standard deviation
TS: Turner Syndrome
WHOQOL-100: World Health Organization Quality of Life 100-items questionnaire
WHOQOL-BREF: World Health Organization Quality of Life 26-items questionnaire
